# ﻿Taxonomic novelty in Pleomonodictydaceae and new reports for *Ampelomycesquisqualis* (Phaeosphaeriaceae), *Melomastiamaolanensis* and *M.oleae* (Pleurotremataceae)

**DOI:** 10.3897/mycokeys.111.135456

**Published:** 2024-12-17

**Authors:** Digvijayini Bundhun, E. B. Gareth Jones, Ruvishika S. Jayawardena, Erio Camporesi, Dhanushka N. Wanasinghe, Indunil C. Senanayake, Vinodhini Thiyagaraja, Kevin D. Hyde

**Affiliations:** 1 Key Laboratory of Phytochemistry and Natural Medicines, Kunming Institute of Botany, Chinese Academy of Sciences, Kunming, Yunnan 650201, China; 2 School of Science, Mae Fah Luang University, Chiang Rai 57100, Thailand; 3 Center of Excellence in Fungal Research, Mae Fah Luang University, Chiang Rai 57100, Thailand; 4 Department of Botany and Microbiology, College of Science, King Saud University, P.O. Box 2455, Riyadh 11451, Saudi Arabia; 5 Gruppo Micologico Forlivese “Antonio Cicognani”, Via Roma 18, Forli, Italy; 6 A.M.B, Circolo Micologico “Giovanni carini”, C.P. 314, Brescia, Italy; 7 Center for Mountain Futures, Kunming Institute of Botany, Chinese Academy of Sciences, Honghe County 654400, China; 8 Department of Soil Science, College of Food and Agriculture Sciences, King Saud University, P.O. Box 145111, Riyadh 11362, Saudi Arabia; 9 Germplasm Bank of Wild Species & Yunnan Key Laboratory for Fungal Diversity and Green Development, Kunming Institute of Botany, Chinese Academy of Sciences, Kunming, 650201, China

**Keywords:** 2 new taxa, Dothideomycetes, Dyfrolomycetales, fungal diversity, morpho-phylogeny

## Abstract

This study introduces a novel genus *Robiniigena*, with its type *R.hyalinospora*. The specimen was collected on dead aerial branches of *Robiniapseudoacacia* in Italy. Based on the examination of morphology and the results of phylogenetic analyses involving nuclear 18S rDNA (SSU), nuclear 28S rDNA (LSU), nuclear rDNA ITS1-5.8S-ITS2 (ITS), translation elongation factor 1-alpha (*tef1-α*) and RNA polymerase II second largest subunit (*rpb2*) sequences, *Robiniigena* is referred to the family Pleomonodictydaceae (Pleosporales). It is characterized by immersed to erumpent, ostiolate ascomata, filiform, septate and cellular pseudoparaphyses, bitunicate, clavate to cylindric-clavate asci and fusiform, hyaline ascospores surrounded by a mucilaginous sheath. This research also establishes the taxonomic placement of the previously unclassified *Inflatispora* (Pleosporales genus *incertae sedis*) within the Pleomonodictydaceae. The sexual morph of *Ampelomycesquisqualis* (Phaeosphaeriaceae) is described for the first time and it is characterized by immersed, perithecial ascomata, a peridium comprising two layers, branched, septate and filiform pseudoparaphyses, short-pedicellate, bitunicate asci with an ocular chamber and sub-hyaline, fusiform, septate ascospores. This species, previously known only in its asexual morph, has been found as a saprobe on *Sonchus* sp. in Italy. Our identification of the sexual morph was based on LSU rDNA and ITS rDNA sequence data. *Melomastiamaolanensis* (Pleurotremataceae) is reported for the first time in Thailand, collected from *Chromolaenaodorata*, while *M.oleae* is documented as a new record from *Durantaerecta* in Thailand.

## ﻿Introduction

The family Pleomonodictydaceae was established to accommodate *Pleomonodictys*, along with two monodictys-like taxa, which formed a distinct clade within the suborder Massarineae, Pleosporales (Hernández-Restrepo et al. 2017). *Pleomonodictys* is characterized by mononematous, micronematous to semi-macronematous conidiophores, which are sometimes reduced to intercalary and polyblastic conidiogenous cells. The conidia, which vary in shape, are blastic, pleurogenous, muriform, and verrucose to tuberculate, and they are often solitary or occur in short chains (Hernández-Restrepo et al. 2017; Bao et al. 2021). The second genus, *Pleohelicoon* was established in the family to accommodate *Helicoon* taxa, which belonged to Pleosporales (Jayasiri et al. 2019). *Pleohelicoon* taxa are characterized by macronematous, mononematous, unbranched and hyaline to brown conidiophores, monoblastic and terminal conidiogenous cells and pale brown to dark fuscous conidia with multi-septate conidial filaments coiled 7–9 times in 3 planes, giving the conidia different shapes (Jayasiri et al. 2019). It is worth noting that no sexual morph has been reported for these genera.

*Inflatispora* was introduced by Zhang et al. (2011) to include the only taxon and type species, *I.pseudostromatica*, characterized by solitary to aggregated, semi-immersed or erumpent ascomata which form under a pseudostroma and open through a broad, round pore, narrow cellular pseudoparaphyses, bitunicate, cylindric-clavate asci, and hyaline, 3-septate, fusiform to cylindrical ascospores with an enlarged upper central cell and surrounded by a mucilaginous sheath (Zhang et al. 2011). *Inflatispora* shares a morphological resemblance to *Nodulosphaeria*, in terms of the swollen upper central cell of the ascospores. However, ascospores of *Nodulosphaeria* have terminal appendages, while in *Inflatisporapseudostromatica*, ascospores are surrounded by a sheath (Zhang et al. 2011). Moreover, phylogenetic analyses of nuclear 28S rDNA (LSU), nuclear 18S rDNA (SSU) and RNA polymerase II second largest subunit (*rpb2*) sequence data of *I.pseudostromatica* showed it grouped with taxa in the Massarineae, forming a separate basal lineage (Zhang et al. 2011). It was however treated as genus *incertae sedis* in the suborder owing to its uncertain familial placement (Zhang et al. 2011). A second species, *Inflatisporacaryotae*, was introduced in the genus based on morpho-phylogenetic analyses (Tibpromma et al. 2017).

*Ampelomycesquisqualis* belongs to Phaeosphaeriaceae (Pleosporales), and is a parasite and biocontrol agent of powdery mildew (Kiss et al. 2004; Liyanage et al. 2018; Manjunatha et al. 2020). This taxon parasitizes the powdery mildew taxa by penetrating hyphae and producing pycnidia in the hyphae, conidiophores, and ascomata of the powdery mildew host, which eventually interferes with the latter’s development (Kiss et al. 2004; Liyanage et al. 2018). *Ampelomycesquisqualis* is characterized by pale to dark brown, pycnidial conidiomata, with walls which are angular textured, and hyaline, ovate to elliptical, aseptate and smooth-walled conidia (Manjunatha et al. 2020). The sexual morph of the species has not been reported so far.

*Melomastia*, typified by *M.mastoidea* (= *M.friesii*), has been subjected to several taxonomic revisions before being finally accommodated in Dothideomycetes (Pleurotremataceae, Dyfrolomycetales) (Barr 1989, 1994; Lumbsch and Huhndorf 2010; Maharachchikumbura et al. 2015, 2016; Norphanphoun et al. 2017; Wijayawardene et al. 2017). It was earlier accommodated in Clypeosphaeriaceae (Sordariomycetes) and was later transferred to Pleurotremataceae, the latter family then assigned to Xylariales (Barr 1989, 1994). However, the genus was then excluded from the family and classified as Ascomycota, genera *incertae sedis* (Kirk et al. 2001; Lumbsch and Huhndorf 2010). While reviewing the backbone tree for Sordariomycetes, Maharachchikumbura et al. (2015) placed *Melomastia* in Sordariomycetes genera *incertae sedis*, while Pleurotremataceae (with the single genus *Pleurotrema*) was accommodated in Chaetosphaeriaceae, Chaetosphaeriales. However, Maharachchikumbura et al. (2016) later excluded Pleurotremataceae from Sordariomycetes, since it was regarded as an earlier name of the family Dyfrolomycetaceae (Dothideomycetes) based on morphology. Meanwhile, *Melomastia* was still considered as an *incertae sedis* genus in Sordariomycetes (Maharachchikumbura et al. 2016). Norphanphoun et al. (2017) finally introduced sequence data for the *Melomastia* taxon, *M.italica* for the first time and showed that *Melomastia* belongs to Dothideomycetes, in the family Pleurotremataceae, along with *Dyfrolomyces* and *Pleurotrema*. They also synonymized *Dyfrolomycesmaolanensis*, a saprobe collected from China (Zhang et al. 2017), to *Melomastiamaolanensis*, owing to its closer phylogenetic affinity with *M.italica* (Norphanphoun et al. 2017). Li et al. (2022) synonymized *Dyfrolomyces* under *Melomastia* based on morphological and phylogenetic analyses. Ascospore septation, which was considered as the principal differentiating character between *Dyfrolomyces* (multi-septate) and *Melomastia* (2-septate), was no longer considered a key for delimitation of the two genera (Li et al. 2022). Kularathnage et al. (2023), however, recently reinstated *Dyfrolomyces* in Pleurotremataceae.

### ﻿Aims of study

In an ongoing survey on the fungal diversity in Italy and Thailand (Chaiwan et al. 2021; Manawasinghe et al. 2022), we recovered several taxa of Dothideomycetes. We herein introduce a new genus *Robiniigena* and accommodate the *Inflatispora* genus *incertae sedis* in Pleomonodictydaceae based on morpho-phylogenetic evidence. Furthermore, the sexual morph of *Ampelomycesquisqualis* is described for the first time, based on LSU–ITS sequence data. We also introduce two new collections of *Melomastia*. *Melomastiamaolanensis* is reported as a new geographical record from Thailand, while *M.oleae*, which was initially introduced from *Oleaeuropaea* in China (Li et al. 2022), is a new host and geographical record on *Durantaerecta* in Thailand based on morphology and phylogeny. Findings herein indicate that there is still much to be discovered vis-a-vis the diversity of Dothideomycetes. Future studies in this class will undoubtedly result in further important taxonomic discoveries and advances.

## ﻿Materials and methods

### ﻿Sample collection, specimen examination, and isolation

Dead stems and twigs bearing fungal fruiting bodies were collected from the terrestrial environment in Italy during the dry (June) and wet (November) seasons, and in Thailand during the wet (July) and dry (November) seasons. The samples were taken to the laboratory in a plastic Ziplock bag and stored inside paper envelopes. Specimens were externally examined with a Motic SMZ 168 stereomicroscope. Free-hand sections of the sporocarps were prepared and placed on water-mounted glass slides. They were then examined using a Nikon ECLIPSE 80i compound microscope with differential interference contrast (DIC) illumination. Microscopic photography was conducted for the ascomata, peridium, pseudoparaphyses, asci, and ascospores. The images were captured with a Canon EOS 750D digital camera fitted to the microscope. Measurements were made with the Tarosoft (R) Image Frame Work v. 0.9.7. using the necessary calibration values and images used for figures were processed with Adobe Photoshop CS6 Extended v. 13.0.1 software (Adobe Systems, San Jose, California).

Single spore isolation was carried out following the method of Senanayake et al. (2020). Ascospore suspensions were made and spread on potato dextrose agar (PDA; 39 g/L) or water agar (WA; 20 g/L). The germinated ascospores were examined after 24 h, after which they were transferred to PDA media. Cultures were incubated at 25 °C in the dark and colony color was determined according to Rayner (1970) after 2–3 weeks of growth on PDA. Herbarium specimens were deposited in Mae Fah Luang University Herbarium (MFLU) while, the living cultures at the Mae Fah Luang University Culture Collection (MFLUCC) in Thailand. Index Fungorum and Faces of Fungi numbers are provided as per Index Fungorum (https://www.indexfungorum.org) and Jayasiri et al. (2015). Taxa descriptions and notes are also uploaded on the GMS microfungi website (https://gmsmicrofungi.org/) (Chaiwan et al. 2021).

### ﻿DNA extraction, PCR amplification, and sequencing

Genomic DNA was extracted from mycelium or directly from fungal fruiting bodies. Fresh mycelium was scraped from cultures grown on PDA at 25 °C for two weeks. Around 15–20 ascomata were removed from the sterilized plant materials using fine sterile needles, while being observed through the stereomicroscope, and placed in 1.5 ml micro-centrifuge tubes (Wanasinghe et al. 2018). DNA was extracted using the Omega Bio-tek kit according to the manufacturer’s instructions. The nuclear 18S rDNA (SSU), nuclear 28S rDNA (LSU), nuclear rDNA ITS1-5.8S-ITS2 (ITS), translation elongation factor 1-alpha (*tef1-α*), and the RNA polymerase II second largest subunit (*rpb2*) were amplified (Table 1). The total volume of the PCR reaction was 25 µL, and it consisted of 12.5 μL of 2× Power Taq PCR MasterMix, 1 μL of each primer, 2 μL genomic DNA extract, and 8.5 μL double-distilled water (ddH_2_O). The PCR protocols were adapted accordingly (Table 1). Thirty-five cycles were used for the denaturation, annealing, and extension steps for each locus (except for *tef1-α* and *rpb2*, where 40 cycles were used). All the PCR thermal cycles included a final extension of 72 °C for 10 mins and a final hold at 4 °C.

**Table 1.** Loci, primers, and PCR amplification conditions used in the present study.

Loci	Primers (forward/ reverse)	Initial Denaturation	Denaturation	Annealing	Extension	References
SSU	NS1/NS4	94 °C, 3 mins	94 °C, 30s	54 °C, 50s	72 °C, 1.30 mins	White et al. (1990)
LSU	LR0R/LR5	95 °C, 5 mins	95 °C, 45s	53 °C, 45s	72 °C, 2 mins	Vilgalys and Hester (1990)
ITS	ITS5/ITS4	95 °C, 5 mins	95 °C, 45s	53 °C, 45s	72 °C, 2 mins	White et al. (1990)
*tef1-α*	983F/2218R	94 °C, 5 mins	94 °C, 30s	58 °C, 1.30 mins	72 °C, 1.20 mins	Carbone and Kohn (1999)
*rpb2*	fRPB2-5f /fRPB2-7cR	95 °C, 5 mins	95 °C, 1 min	54 °C, 2 mins	72 °C, 1.30 mins	Liu et al. (1999), Sung et al. (2007)

PCR products were verified by staining with FluoroDye™ DNA Fluorescent Loading Dye on 1% agarose electrophoresis gels. They were then purified and sequenced using the same primers at the Tsingke Biotech Co. Ltd., Kunming, China. The quality of the sequences obtained was confirmed by checking the chromatograms using BioEdit v. 7.0 (Hall 2004), after which the sequences were assembled into contigs using SeqMan v. 7.1.0. The sequence data derived in this study have been deposited in GenBank (Table 2). New species are established based on recommendations provided by Jeewon and Hyde (2016) and Maharachchikumbura et al. (2021).

**Table 2.** Taxa used for the phylogenetic analyses in the present study, and their corresponding GenBank accession numbers. Type strains are denoted by ^T^. Sequences derived in this study are shown in bold black.

Taxa	Strain	GenBank accession number				
		SSU	LSU	ITS	*tef1–α*	*rpb*2
*Acrospermumadeanum*	M133	EU940031	EU940104	–	–	–
*Acrospermumcompressum*	M151	EU940012	EU940084	–	–	–
*Acrospermumgraminum*	M152	EU940013	EU940085	–		–
*Ampelomycesquisqualis*	AMP	–	–	OP740825	–	–
*Ampelomycesquisqualis*	CBS 129.79	EU754029	EU754128	KY090653	–	–
*Ampelomycesquisqualis*	BRIP 72107	–	–	MZ054399	–	–
*Ampelomycesquisqualis*	CBS 131.79	–	MH872956	MH861188	–	–
*Ampelomycesquisqualis*	CBS 128.79	–	MH872954	MH861185	–	–
*Ampelomycesquisqualis*	CBS 133.32	–	MH866692	MH866692	–	–
*Ampelomycesquisqualis*	CBS 131.31	–	MH866605	AF035781	–	–
*Ampelomycesquisqualis*	HMLAC05119	–	OL739255	OL739255	–	–
*Ampelomycesquisqualis*	Chillan	–	–	MH997723	–	–
*Ampelomycesquisqualis*	CBS 130.79	–	–	HQ108039	–	–
*Ampelomycesquisqualis*	SMKC 22437	–	–	GQ324146	–	–
*Ampelomycesquisqualis*	SMKC 22438	–	–	GQ324128	–	–
***Ampelomycesquisqualis***	**MFLU 23-0142**	–	**PP751625**	**PP751509**	**PP782197**	–
*Anisomeridiumphaeospermum*	MPN539	JN887374	JN887394	–	JN887418	–
*Anisomeridiumubianum*	MPN94	JN887379	–	–	JN887421	–
*Anthosulcatisporasubglobosa*	MFLUCC 17-2065^T^	MT226705	MT214592	MT310636	MT394649	MT394706
*Aquadictyosporalignicola*	MFLUCC 17-1318^T^	–	MF948629	MF948621	MF953164	–
*Aquastromamagniostiolata*	HHUF 30122^T^	AB797220	AB807510	LC014540	AB808486	–
*Asteromassariapulchra*	CBS 124082	GU296137	GU301800	–	GU349066	GU371772
*Bambusicolaaquatica*	MFLUCC 18-1031^T^	MT864293	MN913710	MT627729	MT954392	MT878462
*Brunneomurisporalonicerae*	KUMCC 18-0157^T^	MK356360	MK356346	MK356373	MK359065	–
*Clypeoloculusakitaensis*	HHUF 27557^T^	AB797253	AB807543	AB809631	AB808519	–
*Didymosphaeriarubi-ulmifolii*	MFLUCC 16-1000	MT226672	MT214555	MT310602	MT394734	–
*Dyfrolomyceschromolaenae*	MFLUCC 17-1434^T^	MT214413	–	–	MT235800	–
*Dyfrolomycestiomanensis*	MFLUCC 13-0440^T^	KC692155	KC692156	–	KC692157	–
*Falciformisporalignatilis*	BCC 21117	GU371834	GU371826	KF432942	GU371819	–
*Flavomycesfulophazii*	CBS 135761^T^	KP184082	KP184040	KP184001	–	–
*Fuscostagonosporacamporesii*	MFLU 16-1362^T^	MN750605	MN750590	MN750611	–	–
*Fuscostagonosporacytisi*	MFLUCC 16-0622^T^	KY770977	KY770978	–	KY770979	–
*Fuscostagonosporasasae*	HHUF 29106^T^	AB797258	AB807548	AB809636	AB808524	–
*Halobyssotheciumestuariae*	MFLUCC 19-0386^T^	MN598868	MN598871	MN598890	MN597050	–
*Halomassarinathalassiae*	BCC 17055	GQ925843	GQ925850	–	–	–
*Helicoon* sp.	JAC9590	–	–	MK432689	–	–
*Helminthosporiummicrosorum*	CBS 136910^T^	KY984427	KY984329	KY984329	KY984448	KY984390
*Inflatisporacaryotae*	MFLUCC 13-0825^T^	KY264751	KY264747	KY264743	–	–
*Inflatisporapseudostromatica*	CBS 123110^T^	JN231132	JN231131	–	–	JN231133
*Kaseifertiacubense*	CBS 680.96	AB797218	AB807508	LC014541	AB808484	–
*Katumotoabambusicola*	HHUF 28661^T^	AB524454	AB524595	LC014560	AB539108	AB539095
*Kazuakitanakayuxiensis*	HKAS 122924^T^	ON009092	ON009108	ON009124	ON009267	ON009290
*Latoruacaligans*	CBS 576.65^T^	–	MH870362	MH858723	–	–
*Lentitheciumfluviatile*	CBS 122367	GU296158	GU301825	–	GU349074	–
*Leptosphaeriadoliolum*	CBS 505.75^T^	GU296159	GU301827	JF740205	GU349069	KY064035
*Leucaenicolaaseptata*	MFLUCC 17-2423^T^	MK347853	MK347963	MK347746	MK360059	MK434891
*Longipedicellatamegafusiformis*	MFLU 21-0062^T^	–	MZ538546	MZ538512	MZ567090	–
*Macrodiplodiopsisdesmazieri*	CPC 24971^T^	–	KR873272	KR873240	–	–
*Massarinacisti*	CBS 266.62^T^	AB797249	AB807539	LC014568	AB808514	–
*Melomastiabeihaiensis*	KUMCC 21-0084 ^T^	MZ727002	MZ726990	–	OK043822	–
*Melomastiaclematidis*	MFLUCC 17-2092^T^	MT226718	MT214607	–	MT394663	–
*Melomastiadistoseptata*	NFCCI: 4377^T^	–	MH971236	–	–	–
*Melomastiadistoseptata*	MFLUC 21-0102	–	MT860427	–	–	–
*Melomastiafulvicomae*	MFLUCC 17-2083^T^	MT226719	MT214608	–	MT394664	–
*Melomastiafusispora*	CGMCC 3.20618	OK623494	OK623464	–	OL335189	–
*Melomastiafusispora*	UESTCC 21.0001	OK623495	OK623465	–	OL335190	–
*Melomastiaitalica*	MFLUCC 15-0160^T^	MG029459	MG029458	–	–	–
*Melomastialoropetalicola*	ZHKUCC 22-0174 ^T^	OQ379411	OQ379412	–	–	–
*Melomastiamaolanensis*	GZCC 16-0102^T^	KY111906	KY111905	–	KY814762	–
***Melomastiamaolanensis***	**MFLU 23-0143**	**PP751954**	**PP751616**	–	**PP782198**	–
*Melomastianeothailandica*	MFLU 17-2589^T^	–	MN017857	–	–	–
*Melomastiaoleae*	CGMCC 3.20619^T^	OK623496	OK623466	–	OL335191	–
*Melomastiaoleae*	UESTCC 21.0003	OK623497	OK623467	–	OL335192	–
*Melomastiaoleae*	UESTCC 21.0005	OK623498	OK623468	–	OL335193	–
*Melomastiaoleae*	UESTCC 21.0006	OK623499	–	–	OL335194	–
***Melomastiaoleae***	**MFLUCC 23-0086**	**PP751953**	**PP751621**	–	**PP782199**	–
*Melomastiaphetchaburiensis*	MFLUCC 15-0951^T^	MF615403	MF615402	–	–	–
*Melomastiapuerensis*	ZHKUCC 23-0802 ^T^	OR922340	OR922309	–	OR966284	–
*Melomastiapuerensis*	ZHKUCC 23-0803	OR922341	OR922310	–	OR966285	–
*Melomastiapyriformis*	ZHKUCC 22-0175	OP739334	OP791870	–	OQ718392	–
*Melomastiarhizophorae*	BCC 15481	KF160009	–	–	–	–
*Melomastiarhizophorae*	JK 5456A	GU479766	GU479799	–	GU479860	–
*Melomastiaseptata*	MFLUCC 22-0112^T^	–	OP749870	–	OP760198	–
*Melomastiasichuanensis*	CGMCC 3.20620	OK623500	OK623469	–	OL335195	–
*Melomastiasichuanensis*	UESTCC 21.0008	OK623501	OK623470	–	OL335196	–
*Melomastiasinensis*	MFLUCC 17-1344^T^	MG836700	MG836699	–	–	–
*Melomastiasinensis*	MFLUCC 17-2606	–	OL782048	–	OL875098	–
*Melomastiathailandica*	MFLUCC 15-0945^T^	KX611367	KX611366	–	–	–
*Melomastiathamplaensis*	AND12	OL700222	OL457709	–	–	–
*Melomastiathamplaensis*	MFLUCC 15-0635^T^	KX925436	KX925435	–	KY814763	–
*Melomastiawinteri*	CGMCC 3.20621	OK623502	OK623471	–	OL335197	–
*Montagnulacamporesii*	MFLUCC 16-1369^T^	MN401744	MN401742	MN401746	MN397908	MN397909
*Morosphaeriavelatispora*	PUFD25	MK026765	MK026764	MK026766	MN532688	MN532683
*Muyocoproncastanopsis*	MFLUCC 14-1108	KU726968	KU726965	–	MT136753	–
*Muyocoprondipterocarpi*	MFLU 17-2608	KU726969	KU726966	–	MT136754	–
*Muyocopronheveae*	MFLUCC 17-0066^T^	MH986828	MH986832	–	–	–
*Muyocopronlithocarpi*	MFLUCC 14-1106^T^	KU726970	KU726967	–	MT136755	–
*Neoaquastromabauhiniae*	MFLU 17-0002^T^	MH023315	MH023319	MH025952	MH028247	MH028251
*Neohelicascusaquaticus*	MFLUCC 10-0918	KC886638	KC886640	KC886639	–	–
*Neokalmusiajonahhulmei*	KUMCC 21-0818^T^	ON007048	ON007039	ON007043	ON009133	ON009137
*Neosetophomalonicerae*	KUMCC 18-0155^T^	MK356363	MK356349	MK356375	MK359067	–
*Palawaniathailandensis*	MFLICC 14-1121	–	KY086494	–	–	–
*Palawaniathailandensis*	MFLU 16-1873	KY086495	KY086493	–	–	–
*Palmiascomagregariascomum*	MFLUCC 11-0175	KP753958	KP744495	KP744452	–	KP998466
*Parabambusicolabambusina*	KT 2637	AB797248	AB807538	LC014580	AB808513	–
*Periconiacortaderiae*	MFLUCC 15-0457^T^	KX986345	KX954401	KX965732	KY310703	–
*Periconiapseudodigitata*	KT1395^T^	AB797274	AB807564	LC014591	AB808540	–
*Pleohelicoonfagi*	MFLUCC 17-2538	MK347925	MK348036	MK347816	–	MK434851
*Pleohelicoonfagi*	MFLUCC 15-0182^T^	MK347926	MK348037	MK347817	–	MK434853
*Pleohelicoonrichonis*	CBS 282.54	AY856952	–	MH857332	–	–
*Pleomonodictyscapensis*	DLUCC:1323	–	MZ420757	MZ420742	–	MZ442696
*Pleomonodictyscapensis*	HR 1	AB797261	AB807551	LC014570	AB808527	–
*Pleomonodictyscapensis*	CBS 968.97^T^	–	KY853521	KY853460	–	–
*Pleomonodictysdescalsii*	FMR_12716^T^	–	KY853522	KY853461	–	–
*Pseudoasteromassariaaquatica*	MFLUCC 18-1397^T^	MT864322	MN913721	MT627674	MT954378	–
*Pseudochaetosphaeronemakunmingense*	KUMCC 19-0215^T^	MN792814	MN792815	MN792812	MN794017	–
*Pseudochaetosphaeronemalarense*	CBS 640.73^T^	KF015652	KF015611	KF015656	KF015684	KF015706
*Pseudocoleophomacalamagrostidis*	KT3284^T^	LC014604	LC014609	LC014592	LC014614	–
*Pseudosplanchnonemaphorcioides*	CBS 122935	KY984434	KY984360	KY984360	KY984467	KY984418
*Pseudoxylomyceselegans*	KT 2887	AB797308	AB807598	LC014593	AB808576	–
***Robiniigenahyalinospora***	**MFLU 23-0141^T^**	**PP759387**	**PP759385**	**PP759383**	**PP782200**	**PP476210**
***Robiniigenahyalinospora***	**MFLUCC 23-0074^T^**	**PP759386**	**PP759384**	**PP751493**	**PP782201**	**PP476211**
*Stemphyliumvesicarium*	CBS 191.86^T^	DQ247812	DQ247804	MH861935	DQ471090	KC584471
*Stigmatodiscuslabiatus*	CBS 144700^T^	MH756065	MH756065	–	MH756083	–
*Stigmatodiscusoculatus*	CBS 144Z01^T^	–	MH756069	–	MH756086	–
*Stigmatodiscuspruni*	CBS 142598	KX611110	KX611110	–	KX611111	–
*Submersisporavariabilis*	MFLUCC 17-2360^T^	MT864310	MN913682	–	–	–
*Sulcatisporaacerina*	KT2982^T^	LC014605	LC014610	LC014597	LC014615	–
*Trematosphaeriagrisea*	CBS 332.50^T^	KF015641	KF015614	KF015662	KF015698	KF015720
*Triseptatasexualis*	MFLUCC 11-0002^T^	MN977850	MN977833	MN977832	–	–
*Vikalpagrandispora*	KUNCC 22-12425^T^	OP526628	OP526648	OP526638	OP542240	–

### ﻿Sequence alignment and phylogenetic analyses

Generated SSU, LSU, ITS, *tef1-α* and *rpb2* sequences were subjected to BLASTn searches (https://blast.ncbi.nlm.nih.gov) and similar sequences were downloaded from GenBank based on BLAST similarities and following relevant papers (Tanaka et al. 2015; Phukhamsakda et al. 2016; Hongsanan et al. 2020; Tennakoon et al. 2020; Li et al. 2022; Hyde et al. 2023; Kularathnage et al. 2023; Table 2). Individual gene matrices were aligned using the default setting in MAFFT v. 7.036 (http://mafft.cbrc.jp/alignment/server/) (Katoh et al. 2019) and manually adjusted when necessary in BioEdit v. 7.0 (Hall 2004).

Maximum likelihood (ML) and Bayesian posterior probability (BYPP) analyses were conducted using individual and combined loci datasets. The sequence alignments were converted from FASTA into PHYLIP format using the ALTER (alignment transformation environment, http://www.singgroup.org/ALTER/) bioinformatics web tool (Glez-Peña et al. 2010) before conducting the ML analyses. The latter were then performed in RAxML-HPC2 on XSEDE (v. 8.2.10) (Stamatakis 2014) with the GTRGAMMA substitution model and bootstrapping with 1000 replicates in the CIPRES Science Gateway (Miller et al. 2010) to generate ML trees.

The BYPP analyses were generated using Markov Chain Monte Carlo sampling in MrBayes v. 3.1.2 (Huelsenbeck and Ronquist 2001; Zhaxybayeva and Gogarten 2002). MrModeltest 2.3 (Nylander 2004) was initially used to estimate the best evolutionary models for each locus under the Akaike Information Criterion (AIC) implemented in PAUP v. 4.0b10 (Swofford 2002). Six simultaneous Markov chains were then run for the necessary number of generations, as mentioned under the phylogenetic trees. Trees were sampled every 1000^th^ generation. Convergence was declared when the standard deviation of split frequencies was less than 0.01. The first 20% of generated trees, representing the burn-in phase, were discarded. The remaining 80% of trees were used to calculate the posterior probabilities in the majority rule consensus tree.

The resulting trees were viewed in FigTree v. 1.4.0 (Rambaut 2012) and modified in Microsoft PowerPoint. The ML bootstrap values (ML BS) equal to or greater than 60% and BYPP equal to or greater than 0.95 are given above or below the branches in each phylogenetic tree. The final alignments were deposited in TreeBASE, with the submission IDs mentioned under the respective phylogenetic trees.

## ﻿Results

### ﻿Phylogenetic analyses

Analysis 1

**Figure 1.**
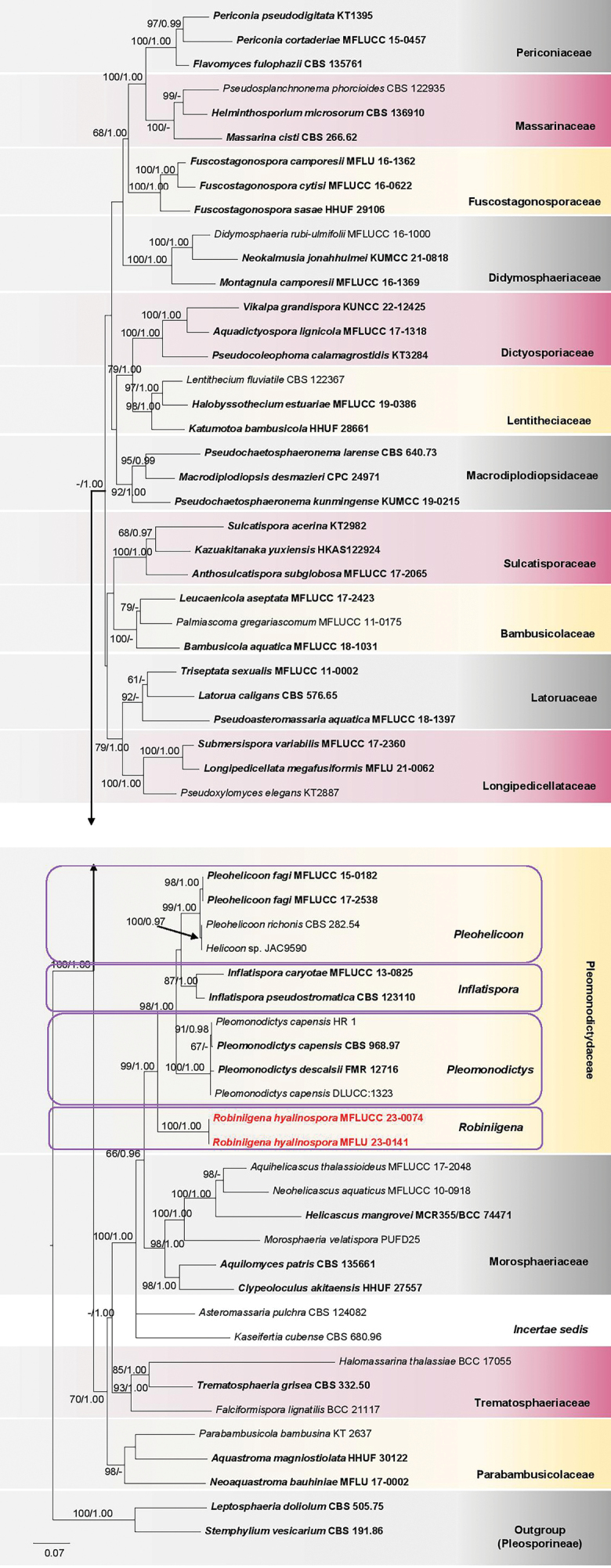
Phylogram generated from maximum likelihood analysis based on SSU–LSU–ITS– *tef1-α*–*rpb2* combined dataset for the families in Massarineae. The evolutionary model GTR+I+G was applied as the best-fit model for all the loci. The BYPP analysis was run for 3 million generations. The tree is rooted with *Leptosphaeriadoliolum* (CBS 505.75) and *Stemphyliumvesicarium* (CBS 191.86). Maximum likelihood bootstrap, ML BS (≥60%), and BYPP (≥0.95) supports are respectively shown above or below the branches. Type strains are in bold black, and the novel taxon is in bold red. (-) indicate ML BS < 60% or BYPP < 0.95. TreeBASE submission ID: 31443 (Reviewer access URL: http://purl.org/phylo/treebase/phylows/study/TB2:S31443?x-access-code=8ac9bd9583064274a7dded00c2fa3595&format=html)

The combined SSU–LSU–ITS–*tef1-α*–*rpb2* dataset for the suborder Massarineae comprised 61 strains, including the strains MFLU 23-0141 and MFLUCC 23-0074 collected in the present study and the outgroup taxa *Leptosphaeriadoliolum* (CBS 505.75) and *Stemphyliumvesicarium* (CBS 191.86). The concatenated alignment consisted of 5220 characters. The ML and BYPP trees were almost similar in topology and did not differ significantly. The best RAxML tree with a final likelihood value of -50756.398321 was obtained and is illustrated in Fig. 1. The alignment had 2542 distinct alignment patterns, and 39.0% of gaps or undetermined characters were present. Estimated base frequencies were as follows: A = 0.241009, C = 0.249659, G = 0.270618, T = 0.238714 and the substitution rates were: AC = 1.287337, AG = 3.234605, AT = 1.329219, CG = 1.078417, CT = 6.074542, GT = 1.0. The gamma distribution rate parameter was 0.243991, while the Tree length was 6.343450.

*Robiniigenahyalinospora*MFLU 23-0141 and MFLUCC 23-0074 grouped in Pleomonodictydaceae with 99% ML BS, 1.00 BYPP statistical support (Fig. 1). They were basal in the family, shared by ‘*Helicoon*’ sp. (JAC9590), *Inflatisporacaryotae* (MFLUCC 13-0825), *I.pseudostromatica* (CBS 123110), *Pleohelicoonfagi* (MFLUCC 15-0182, MFLUCC 17-2538), *P.richonis* (CBS 282.54), *Pleomonodictyscapensis* (CBS 968.97, DLUCC:1323, HR 1) and *Pl.descalsii* (FMR 12716). *Inflatisporacaryotae* and *I.pseudostromatica* grouped in a subclade with 87% ML BS, and 1.00 BYPP support values. The two strains of *Inflatispora* are grouped with the three strains of *Pleohelicoon* and the strain ‘*Helicoon*’ sp. (JAC9590) with 54% ML BS statistical support. The four strains of *Pleomonodictys* formed a subclade, sister to the ‘*Helicoon*’ sp., *Inflatispora*, and *Pleohelicoon* taxa.

#### ﻿Analysis 2

**Figure 2.**
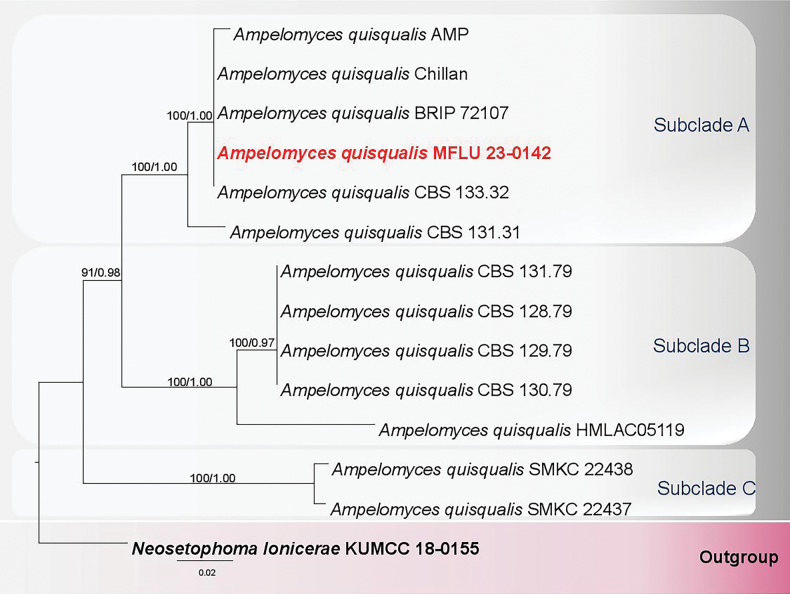
Phylogenetic tree generated from RaxML analysis, based on LSU–ITS matrix for *Ampelomyces*. The evolutionary model GTR+I was applied for LSU, while GTR+I+G was the best-fit model for ITS. The BYPP analysis was run for 1 million generations. The tree is rooted with *Neosetophomalonicerae* (KUMCC 18-0155). Maximum likelihood bootstrap, ML BS (≥60%), and BYPP (≥0.95) supports are shown above the branches, respectively. The type strain is in bold, and the newly collected strain is in bold red. TreeBASE submission ID: 31444.

The LSU–ITS matrix for *Ampelomyces* comprised 14 strains, including our strain MFLU 23-0142 and the outgroup taxon *Neosetophomalonicerae* (KUMCC 18-0155). The concatenated alignment consisted of 1360 characters. The ML and BYPP trees were similar in topology and did not differ significantly. The best RAxML tree with a final optimization likelihood value of -3245.931378 was yielded and is presented below (Fig. 2). The alignment had 178 distinct patterns and 27.7% of gaps or undetermined characters. Estimated base frequencies were as follows: A = 0.247457, C = 0.216767, G = 0.254577, T = 0.281199 and the substitution rates were: AC = 4.277905, AG = 11.081952, AT = 4.397096, CG = 3.194723, CT = 27.614052, GT = 1.0. The gamma distribution rate parameter was 0.265280 while the Tree-length was 0.291945.

Strain MFLU 23-0142 nested with *Ampelomycesquisqualis* (AMP, Chillan, BRIP 72107 and CBS 133.32) in a subclade (A) with 100% ML BS, 1.00 BYPP support (Fig. 2).

#### ﻿Analysis 3

**Figure 3.**
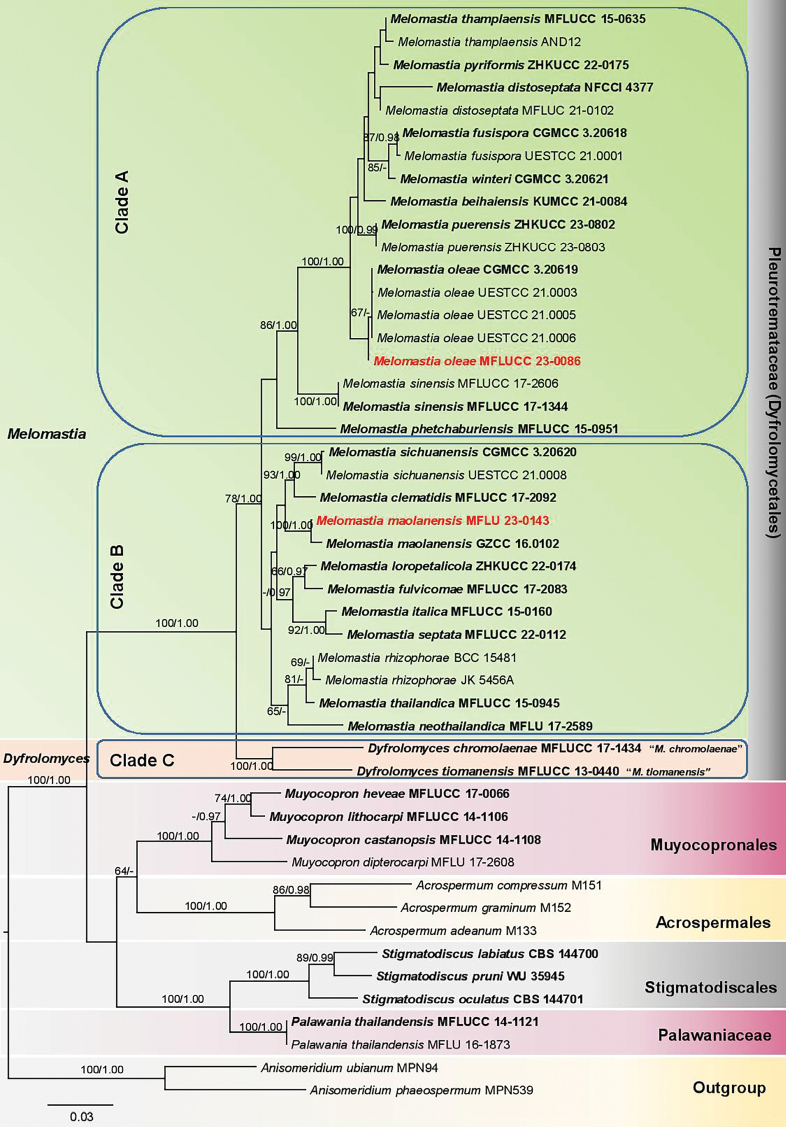
Phylogram generated from maximum likelihood RaxML analysis based on combined SSU, LSU, and *tef1-α* sequence data. The evolutionary model SYM+I+G was chosen as the best-fit model for SSU while GTR+I+G was applied for LSU and *tef1-α*. The BYPP analysis was run for 2 million generations. The tree is artificially rooted with *Anisomeridiumphaeospermum* (MPN539) and *A.ubianum* (MPN94). Maximum likelihood bootstrap, ML BS (≥60%), and BYPP (≥0.95) are given respectively above or below the branches. Type strains are in bold. The newly generated sequences are indicated in bold red. (-) indicates ML BS < 60% or BYPP < 0.95. TreeBASE submission ID: 31445.

An SSU–LSU–*tef1-α* dataset for Dyfrolomycetales and related orders and families, consisting of 48 strains, was used for analyzing the two strains MFLU 23-0143 and MFLUCC 23-0086 collected in the present study. The outgroup taxa used were *Anisomeridiumphaeospermum* (MPN539) and *A.ubianum* (MPN94). The ML and BYPP trees were almost similar in topology and did not differ significantly. The best RaxML tree with a final likelihood value of -13964.812497 was yielded and is presented below (Fig. 3). The matrix had 976 distinct alignment patterns, with 23.70% of gaps or undetermined characters. Estimated base frequencies were as follows: A = 0.241398, C = 0.259224, G = 0.291514, T = 0.207864 and the substitution rates were: AC = 0.884199, AG = 2.067368, AT = 1.098051, CG = 0.895791, CT = 7.809461, GT = 1.0. The gamma distribution rate parameter was 0.210247 while the Tree-length was 1.259771.

The strain MFLU 23-0143 clustered with *Melomastiamaolanensis* (GZCC 16-0102) with 100% ML BS, 1.00 BYPP statistical support. The isolate MFLUCC 23-0086 was sister to *M.oleae* (CGMCC3.20619, UESTCC 21.0003, UESTCC 21.0005, UESTCC 21.0006) with 55% ML BS and 0.67 BYPP support values (Fig. 3).

### ﻿Taxonomy

In this section, we follow the classification of Wijayawardene et al. (2022) and revise it as needed. The amended description for Pleomonodictydaceae is provided, along with notes on *Inflatispora*, which is herein accommodated in the family. Descriptions, notes and illustrations are provided for: *Robiniigenahyalinospora* gen. et sp. nov., *Ampelomycesquisqualis*, *Melomastiamaolanensis* and *M.oleae*.

#### ﻿ Pleomonodictydaceae

Taxon classificationFungi﻿Pleomonodictydaceae

Hern.-Restr., J. Mena & Gené, Stud. Mycol. 86: 76 (2017), amended

5EC512B1-4610-5991-8C9C-A159635F7402

Index Fungorum: IF820279

Facesoffungi Number: FoF08344

##### Description.

***Saprobic*** on woody substrates in terrestrial and aquatic habitats. **Sexual morph**: ***Ascomata*** solitary or in groups, often growing under or in a pseudostroma, immersed, semi-immersed to erumpent, perithecial, brown to dark brown, ostiolate. ***Ostiolar neck*** papillate. ***Peridium*** multi-layered, outer layer usually fusing with the stroma or host tissue. ***Pseudoparaphyses*** hyaline, filiform, cellular. ***Asci*** bitunicate, 8-spored, clavate to cylindric-clavate, pedicellate, with an ocular chamber. ***Ascospores*** 1–3-seriate, fusiform to cylindrical, hyaline, septate, surrounded by a mucilaginous sheath. **Asexual morph**: hyphomycetous. See Hernández-Restrepo et al. (2017), Jayasiri et al. (2019), Bao et al. (2021) for further details.

##### Type genus.

*Pleomonodictys* Hern.-Restr., J. Mena & Gené

##### Notes.

Pleomonodictydaceae has so far accommodated the two hyphomycetous genera, *Pleohelicoon* and *Pleomonodictys* (Hernández-Restrepo et al. 2017; Jayasiri et al. 2019), whose sexual morphs are still undetermined. In the present study, *Inflatispora* and the novel genus, *Robiniigena* are accepted in the family based on the combined SSU–LSU–ITS–*tef1-α*–*rpb2* phylogenetic analyses (Fig. 1). Since both genera are known in their sexual morphs, the description of Pleomonodictydaceae is amended to include their sexual morphological characteristics.

#### ﻿ Inflatispora

Taxon classificationFungiPleosporales﻿Pleomonodictydaceae

Y. Zhang ter, J. Fourn. & K.D. Hyde, Sydowia 63(2): 290 (2011)

878D97D6-F4A2-50B1-B116-C9EE2861D8F8

Index Fungorum: IF561844

Facesoffungi Number: FoF11812

##### Description and illustrations.

see in Zhang et al. (2011), Tibpromma et al. (2017)

##### Type species.

*Inflatisporapseudostromatica* Y. Zhang ter, J. Fourn. & K.D. Hyde

##### Notes.

When Tanaka et al. (2015) conducted a revision of the Massarineae, *Inflatisporapseudostromatica* clustered with *Monodictyscapensis* (now synonymized to *Pleomonodictyscapensis*, Hernández-Restrepo et al. 2017) in a clade which was considered as *incertae sedis*. When the second taxon, *I.caryotae* was introduced, it was sister to *I.pseudostromatica*, and together both species clustered with *M.capensis* and *Asteromassariapulchra* (fig. 39 in Tibpromma et al. 2017). None of these taxa had any familial placement and thus, *Inflatispora* was referred to Massarineae*incertae sedis* (Tibpromma et al. 2017). At the same time, in the phylogenetic analyses conducted by Hernández-Restrepo et al. (2017), *Inflatispora* (*I.pseudostromatica*) grouped with taxa of the novel genus *Pleomonodictys* (*Pleomonodictyscapensis* and *Pl.descalsii*) described in that study, and those of *Bactrodesmium* (*B.cubense*), *Clypeoloculus* (*C.akitaensis*) and *Morosphaeria* (*M.velatispora*). However, *Pleomonodictys* was placed in the new family Pleomonodictydaceae, while the other genera were reported as *incertae sedis* (Hernández-Restrepo et al. 2017). Later, when Jayasiri et al. (2019) introduced *Pleohelicoon* in Pleomonodictydaceae, *Inflatispora* clustered with *Pleohelicoon* and *Pleomonodictys* in a clade, where it formed a distinct basal lineage to the two genera with low statistical support and was still considered as *incertae sedis* taxon. In the present study *Inflatispora* clusters in Pleomonodictydaceae with 99% ML BS, 1.00 BYPP statistical support (Fig. 1). *Inflatispora* resembles (in its sexual morph) *Robiniigenahyalinospora* gen. et sp. nov. in Pleomonodictydaceae and is accepted in the family based on morpho-phylogenetic evidence.

#### ﻿ Robiniigena

Taxon classificationFungiPleosporales﻿Pleomonodictydaceae

Bundhun, Camporesi & K.D. Hyde gen. nov.

8736F4C0-9269-561F-86BF-06EA7F2A1BB8

Index Fungorum: IF902255

Facesoffungi Number: FoF14884

##### Etymology:

The name is based on the host genus *Robinia*, from which the fungus was isolated.

##### Description.

***Saprobic*** on woody substrates. **Sexual morph**: ***Ascomata*** immersed, with black dots present on host surface or erumpent, scattered, solitary or growing in groups in a pseudostroma, perithecial, globose to subglobose, usually unilocular, brown to dark brown, ostiolate. ***Ostiolar neck*** papillate, composed of hyaline pseudoparenchymatous cells. ***Peridium*** comprising two regions, outer region multi-layered, composed of brown to dark brown, thick-walled cells of ***textura angularis***; inner layer made up of lightly pigmented to hyaline cells. ***Hamathecium*** composed of hyaline, filiform, cellular, branched, indistinctly septate pseudoparaphyses. ***Asci*** bitunicate, fissitunicate, 8-spored, hyaline, clavate to cylindric-clavate, thin-walled, short-pedicellate, apically rounded, with an ocular chamber. ***Ascospores*** overlapping 1–2-seriate, hyaline, narrow to broadly fusiform, straight to slightly curved, euseptate, constricted at the septum, both ends conically rounded, smooth-walled, usually guttulate, surrounded by a thick or spreading mucilaginous sheath at maturity. **Asexual morph**: Undetermined.

##### Type species.

*Robiniigenahyalinospora* Bundhun, Camporesi & K.D. Hyde

##### Notes.

This monotypic genus forms a basal lineage in Pleomonodictydaceae with 99% ML BS and 1.00 BYPP statistical support (Fig. 1). *Robiniigena* shares several characteristics with *Inflatispora*, in terms of cellular pseudoparaphyses, clavate to cylindric-clavate, short-pedicellate asci, and hyaline, septate ascospores surrounded by a mucilaginous sheath (Zhang et al. 2011; Tibpromma et al. 2017). The ascospores of *Robiniigena* are, however, narrow to broadly fusiform and sometimes contain few large guttules, while those of *Inflatispora* are mostly narrowly fusiform to almost cylindrical and are ornamented with small guttules (Zhang et al. 2011; Tibpromma et al. 2017). The two genera are also phylogenetically distinct, with *Inflatispora* grouping with *Pleohelicoon* and *Pleomonodictys* in a clade and *Robiniigena* forming a basal separate lineage (Fig. 1).

#### ﻿ Robiniigena hyalinospora

Taxon classificationFungiPleosporales﻿Pleomonodictydaceae

Bundhun, Camporesi & K.D. Hyde sp. nov.

07660425-D973-5A8C-8449-595ECB2FB2BA

Index Fungorum: IF902256

Facesoffungi Number: FoF14885

Fig. 4

##### Etymology.

The epithet refers to the hyaline ascospores.

##### Holotype.

MFLU 23-0141

##### Description.

***Saprobic*** on *Robiniapseudoacacia*. **Sexual morph**: ***Ascomata*** 320–470 µm high, 250–600 µm diam. (x– = 396 × 471 µm, n = 5), immersed, with black dots present on host surface or erumpent, visible in bark fissures, scattered, solitary or aggregated in a pseudostroma, perithecial, globose to subglobose, usually unilocular, rarely bilocular, brown to dark brown, coriaceous, ostiolate. ***Ostiolar neck*** 90–110 µm wide, papillate, comprising amorphous hyaline cells. ***Peridium*** 30–50 µm thick near the apex, 20–35 µm wide at the sides and base, comprising two regions; outer region multi-layered, composed of brown to dark brown, thick-walled cells of ***textura angularis***, fusing and becoming indistinguishable from the pseudostroma or host cells towards the outermost side; inner layer made up of lightly pigmented to hyaline cells merging with the pseudoparaphyses. ***Pseudoparaphyses*** 1.5–2.5 µm wide, numerous, filiform, cellular, branched, indistinctly septate, usually guttulate, surrounding the asci and along the innermost layer of the peridium. ***Asci*** 80–145(–160) × 15–25 µm (x– = 111.5 × 20.9 µm, n = 15), bitunicate, fissitunicate, 8-spored, hyaline, clavate to cylindric-clavate, straight to slightly curved, thin-walled, short-pedicellate, apically rounded, with an ocular chamber. ***Ascospores*** (25–)30–40(–45) × 6–10(–12) µm (x– = 36.8 × 8.6 µm, n = 55), overlapping 1–2-seriate, hyaline, narrow to broadly fusiform, straight to slightly curved, 1-euseptate, constricted at the septum, symmetrical or upper cell slightly longer than lower cell, wider upper cell, broad to acute and conically rounded at both ends, smooth-walled, sometimes guttulate, surrounded by a thick or spreading mucilaginous sheath when mature. **Asexual morph**: Not observed.

**Figure 4.**
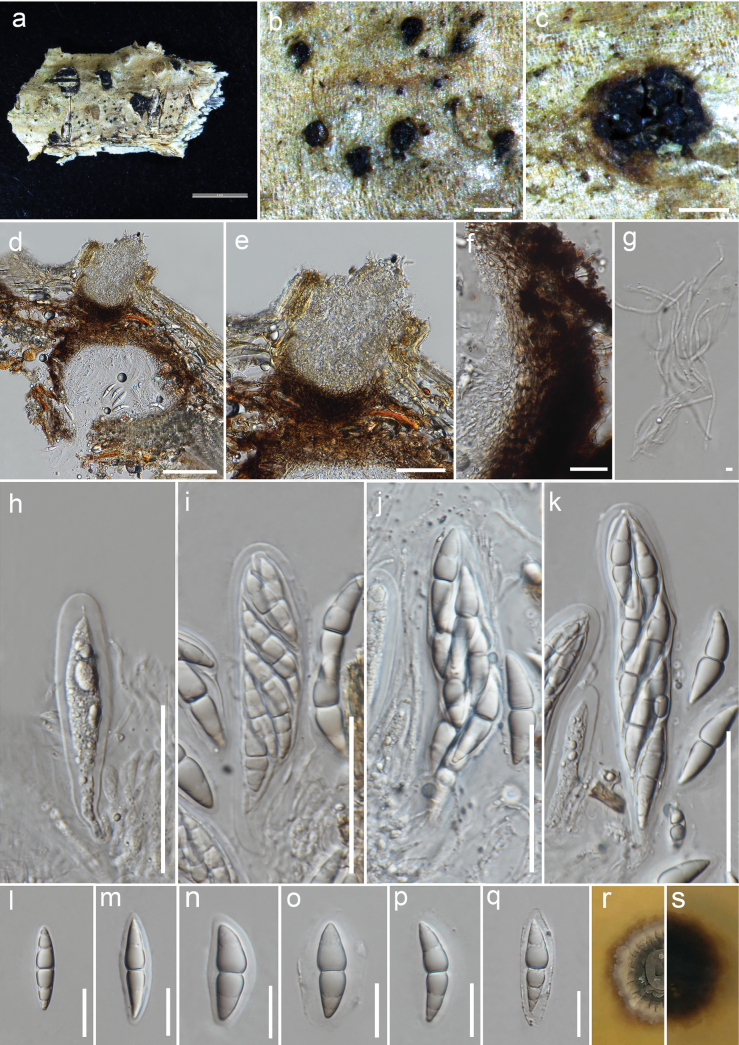
*Robiniigenahyalinospora* (MFLU 23-0141, holotype) **a** host substrate **b, c** ascomata on substrate **d** vertical section of an ascoma **e** section of an ostiolar neck **f** peridium **g** pseudoparaphyses **h–k** immature and mature asci **l–q** immature and mature ascospores **r** upper view of colony **s** reverse view of colony. Scale bars: 200 µm (**b**); 400 µm (**c**); 100 µm (**d**); 50 µm (**e, h–k**); 20 µm (**f, l–q**); 3 µm (**g**).

##### Culture characteristics.

Ascospores germinated on WA within 24 hr. Colony on PDA, reaching 2 cm diam. after 15 days at 25 °C; above view dark grey in the middle and pale grey edges, dense, circular, umbonate, surface rough, radially furrowed, fimbriate; reverse dark brown, radiating outwardly.

##### Material examined.

Italy • Padova, near Torreglia; on dead aerial branches of *Robiniapseudoacacia*, 18 Nov 2021, E. Camporesi IT 4807 (holotype MFLU 23-0141), ex-type culture MFLUCC 23-0074.

##### Notes.

*Robiniigenahyalinospora* resembles *Inflatisporapseudostromatica* by its globose to sub-globose ascomata, short-pedicellate asci, and ascospores whose upper cells are comparatively broader than the lower part (Zhang et al. 2011). The ascomata of *R.hyalinospora* are however, coriaceous and occur in a pseudostroma mainly when they are aggregated, while the ascomata of *I.pseudostromatica* are hard and form under a black pseudostroma both when the ascomata are solitary or occur in groups (Zhang et al. 2011). Furthermore, *R.hyalinospora* has narrow to broadly fusiform, 1-euseptate ascospores with conically rounded ends, whereas *I.pseudostromatica* comprises narrowly fusiform to nearly cylindrical, 3-septate ascospores with broadly or narrowly rounded ends (Zhang et al. 2011).

*Robiniigenahyalinospora* is similar to *I.caryotae*, in terms of 1-(eu)septate ascospores with a constricted middle septum and surrounded by a mucilaginous sheath (Tibpromma et al. 2017). However, *I.caryotae* has immersed ascomata while *R.hyalinospora* has immersed or erumpent ascomata, and the ascospores of *I.caryotae* are narrowly fusiform with acute ends while those of *R.hyalinospora* are narrow to broadly fusiform, with comparatively broader and conically rounded ends. *Robiniigenahyalinospora*, *I.caryotae* and *I.pseudostromatica*, are the only taxa in Pleomonodictydaceae with known sexual morphs.

#### ﻿ Ampelomyces quisqualis

Taxon classificationFungiPleosporales﻿Pleomonodictydaceae

Ces., in Klotzsch, Bot. Ztg. 10: 301 (1852)

D4B15DF2-0BA7-5B87-B5E4-D7FE19F7F7B5

Index Fungorum: IF121267

Facesoffungi Number: FoF11631

Fig. 5

##### Description.

***Saprobic*** on stem of *Sonchus* sp. **Sexual morph**: ***Ascomata*** 140–180 µm high, 200–260 µm diam. (x– = 159 × 221 µm, n = 5), immersed, appearing as black dots on the host surface, solitary to aggregated, scattered, perithecial, unilocular, globose to subglobose, dark brown, ostiolate. ***Ostiole*** centric, comprising hyaline cells. ***Peridium*** 20–30 µm thick near the ostiole, 10–25 µm wide at the sides and 10–20 µm thick at the base, 3–4-layered, outer layer made up of thick-walled, brown cells of ***textura angularis***; inner layer made up of thin-walled, pale brown to hyaline cells of ***textura angularis***. ***Pseudoparaphyses*** 1–1.5 µm wide, numerous, hyaline, filiform, branched, septate, cellular, usually guttulate, surrounding the asci. ***Asci*** 45–65(–68) × 5–7.5 µm (x– = 56.7 × 6.4 µm, n = 40), bitunicate, 8-spored, cylindrical, straight to slightly curved, thin-walled, short-pedicellate, bulbous, with an apical ocular chamber. ***Ascospores*** 12–17 × 2–4 µm (x– = 15.3 × 3 µm, n = 50), overlapping uni- to bi-seriate, hyaline when immature, sub-hyaline on maturity, fusiform, straight to slightly curved, 2-celled with a median septum, symmetrical or upper cell slightly longer than lower cell, cell above septum slightly enlarged and with round or conical ends, lower cell mostly with round ends, minutely guttulate, sometimes both ends containing hyaline appendages which disappear with age. **Asexual morph**: see Manjunatha et al. (2020).

**Figure 5.**
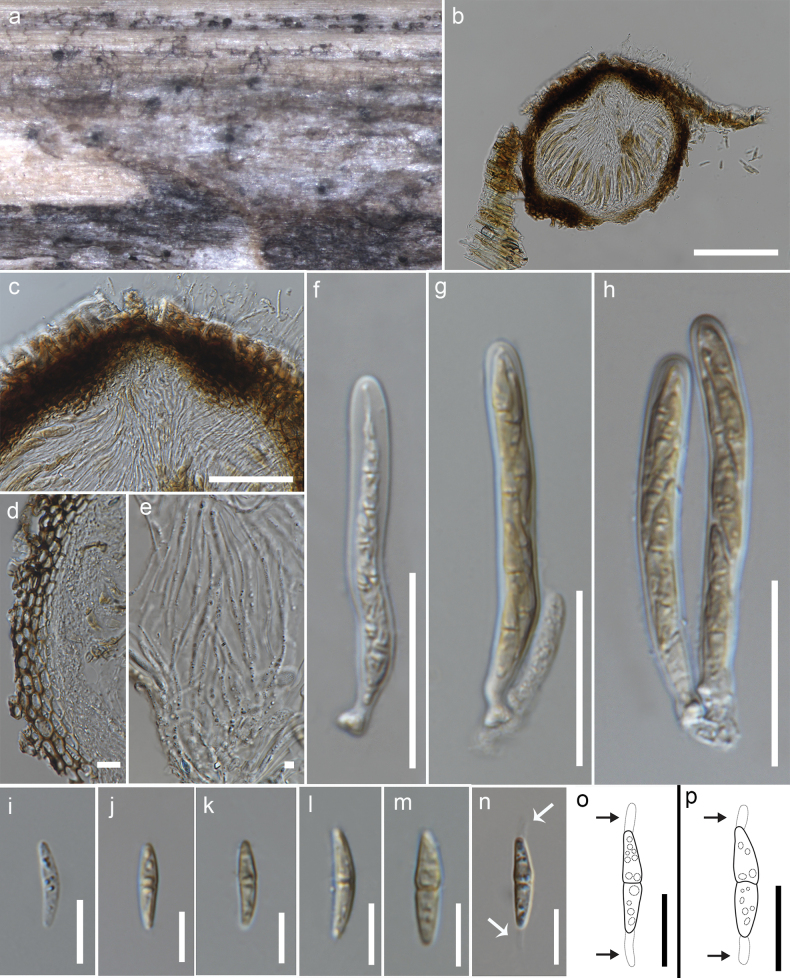
*Ampelomycesquisqualis* (MFLU 23-0142) **a** ascomata on host substrate **b** vertical section of an ascoma **c** section of an ostiole **d** peridium **e** pseudoparaphyses **f–h** asci **i–m** ascospores **n–p** hyaline appendages at both ends of ascospores (shown by arrows). Scale bars: 100 µm (**b**); 50 µm (**c**); 10 µm (**d, i–p**,); 3 µm (**e**); 30 µm (**f–h**).

##### Material examined.

Italy • Forlì-Cesena, Valico Tre Faggi - Premilcuore; on dead aerial stems of *Sonchus* sp., 22 Jun 2021, E. Camporesi IT 4713, Herbarium material MFLU 23-0142.

##### Notes.

Phylogenetic analyses based on the combined LSU–ITS dataset showed that strain MFLU 23-0142 grouped with *Ampelomycesquisqualis* (AMP, Chillan, BRIP 72107, and CBS 133.32) strains with 100% ML BS, 1.00 BYPP support (Fig. 2). While there was no base pair (bp) difference among the three strains of *A.quisqualis* (Chillan, BRIP 72107 and CBS 133.32) and MFLU 23-0142 vis-à-vis the ITS sequence (based on the aligned untrimmed dataset, including gaps), 1.2% (6/518 bp) difference between *A.quisqualis* (AMP) and strain MFLU 23-0142 was observed. Similarly, there was 0.1% bp (1/876 bp) difference between *A.quisqualis* (CBS 133.32) and MFLU 23-0142 with regards to the LSU sequence (no LSU sequence data are available for strains AMP, Chillan, and BRIP 72107 in GenBank). A morphological comparison could not be made since our strain was collected in its sexual morph while *Ampelomyces* has so far been reported in its asexual morph. Therefore, the strain MFLU 23-0142 is described as the sexual morph of *A.quisqualis* based on phylogenetic support.

#### ﻿ Melomastia maolanensis

Taxon classificationFungiPleosporales﻿Pleomonodictydaceae

(Jin F. Zhang, Jian K. Liu, K.D. Hyde & Zuo Y. Liu) Norphoun, T.C. Wen & K.D. Hyde, Cryptog. Mycol. 38(4): 518 (2017)

69FA93C3-C946-5FD4-80B8-2D1970430332

Index Fungorum: IF554041

Facesoffungi Number: FoF02695

Fig. 6

##### Description.

***Saprobic*** on dead stems of *Chromolaenaodorata*. **Sexual morph**: ***Ascomata*** 420–440 µm high (including ostiole), 330–365 µm diam. (x– = 426 × 351.3 µm, n = 5), visible as dark, raised, black spots on host, scattered, solitary to gregarious, immersed, with erumpent ostiole, perithecial, subglobose to obpyriform, coriaceous, dark brown to black, papillate, with a black clypeus. ***Ostiole*** 170–190 × 160–190 µm, central, oblong, dark brown to black, periphysate. ***Peridium*** 18–25 µm wide, comprising two layers; outer layer made up of thick-walled, dark brown cells fusing with host tissue, inner layer composed of thin-walled, hyaline cells. ***Pseudoparaphyses*** 1.5–3 µm wide, numerous, filiform, generally aseptate and unbranched, tapering towards the apex. ***Asci*** 80–125 × 4–7 µm (x– = 102.7 × 5.5 µm, n = 20), 8-spored, non fissitunicate, long cylindrical, slightly flexuous, short-pedicellate, truncate or rounded at the apex, with a small ocular chamber. ***Ascospores*** 14–18.5 × 3–5 µm (x– = 17.2 × 4.2 µm, n = 20), uniseriate, partially overlapping, hyaline, fusiform, with rounded or acute ends, 3-septate, constricted at the septa, smooth-walled, guttulate, surrounded by a mucilaginous sheath which vanishes with age. **Asexual morph**: Not observed.

**Figure 6.**
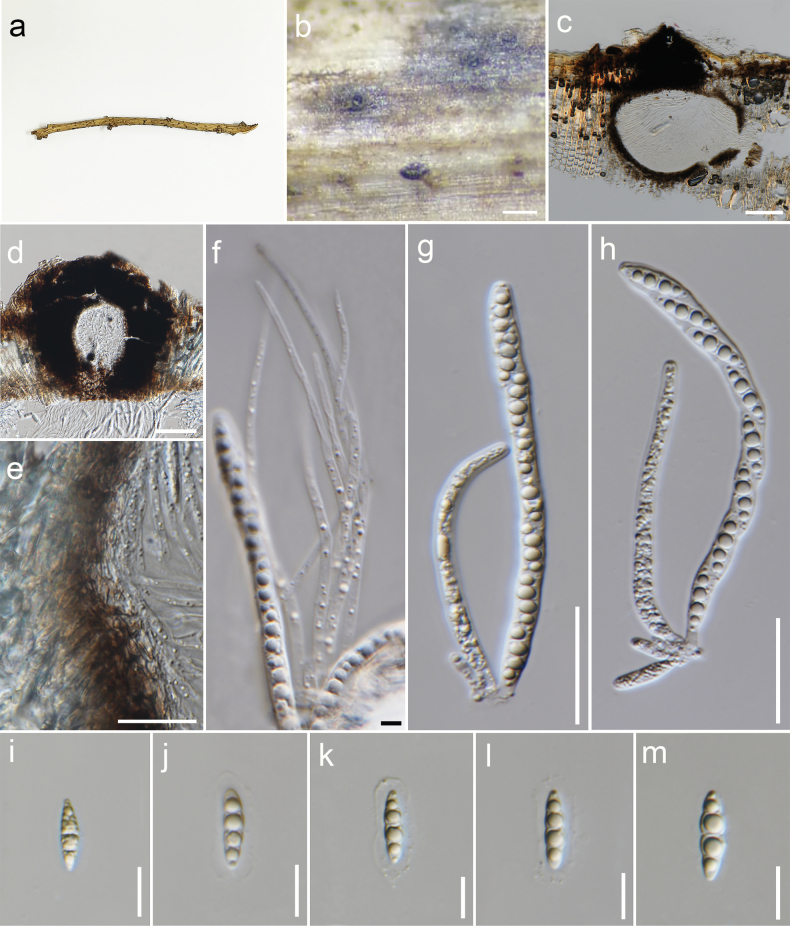
*Melomastiamaolanensis* (MFLU 23-0143) **a** stem of *Chromolaenaodorata***b** ascomata on host **c** vertical section of an ascoma **d** ostiole **e** peridium **f** pseudoparaphyses **g, h** asci **i–m** ascospores. Scale bars: 200 µm (**b**); 100 µm (**c**); 50 µm (**d**); 20 µm (**e**); 5 µm (**f**); 30 µm (**g, h**); 10 µm (**i–m**).

##### Material examined.

Thailand • Chiang Mai, on dead stems of *Chromolaenaodorata*, 7 Jul 2021, N.S. Wijesinghe CMN1, Herbarium material MFLU 23-0143.

##### Notes.

In this study, strain MFLU 23-0143 clustered with *M.maolanensis* (GZCC 16-0102) with 100% ML BS, 1.00 BYPP support in the multi-locus phylogeny (Fig. 3). Nucleotide comparison of LSU and *tef1-α* revealed 0.1% (1/911 bp) and 1.3% (12/921 bp) differences between the two strains (MFLU 23-0143 and GZCC 16-0102), indicating insufficient phylogenetic differences to separate them as two different taxa (Jeewon and Hyde 2016; Maharachchikumbura et al. 2021). The two strains are also morphologically similar with ostiolate and papillate, immersed ascomata, long cylindrical and short-pedicellate asci, and ascospores that are fusiform, guttulate, and 3-septate. Therefore, strain MFLU 23-0143 is identified as *M.maolanensis* based on morphological and phylogenetic evidence. The main morphological difference between the two strains is the presence of a mucilaginous sheath around the ascospores of *M.maolanensis*MFLU 23-0143, while no such report was made for the strain GZCC 16-0102 (Zhang et al. 2017). Variations in the size of the morphological characteristics between the two strains may be accounted for by environmental differences. Zhang et al. (2017) reported *M.maolanensis* from an undetermined tree branch in China. The same taxon is reported here from dead stems of *Chromolaenaodorata* in Thailand.

#### ﻿ Melomastia oleae

Taxon classificationFungiPleosporales﻿Pleomonodictydaceae

W.L. Li, Maharachch. & Jian K. Liu, Journal of Fungi 8(1, no. 76): 10 (2022)

A94CF02E-E848-54B2-9E07-E9AF65738BB4

Index Fungorum: IF841500

Facesoffungi Number: FoF10534

Fig. 7

##### Description.

***Saprobic*** on dead branches of *Durantaerecta*. **Sexual morph**: ***Ascomata*** 415–420 µm high, 500–520 µm diam. (x– = 418.9 × 511.1 µm, n = 5), visible as black, cone-shaped structures on host surface, usually solitary, scattered, semi-immersed to erumpent, globose to ampulliform, carbonaceous, dark brown to black, ostiolate. ***Ostiole*** central, dark brown to black, papillate, carbonaceous, periphysate. ***Peridium*** 18–35 µm diam., comprising two regions in vertical section; outer region carbonaceous, made up of 5–7-layered, thick-walled, brown cells of ***textura angularis*** to ***textura epidermoidea***, innermost region composed of compressed, hyaline cells. ***Pseudoparaphyses*** 1.5–2.5 µm wide, numerous, filiform, unbranched, sometimes septate. ***Asci*** 100–180 × 5.5–8 µm (x– = 142.7 × 6.7 µm, n = 22), 8-spored, non fissitunicate, cylindrical, straight to flexuous, rounded at the apex, with a small ocular chamber, short-pedicellate. ***Ascospores*** 20–25 × 5–6 µm (x– = 22.2 × 5.5 µm, n = 30), uniseriate, hyaline, fusiform, with rounded or obtuse ends, 3-septate, slightly constricted at the septa, guttulate, smooth-walled. **Asexual morph**: Not observed.

**Figure 7.**
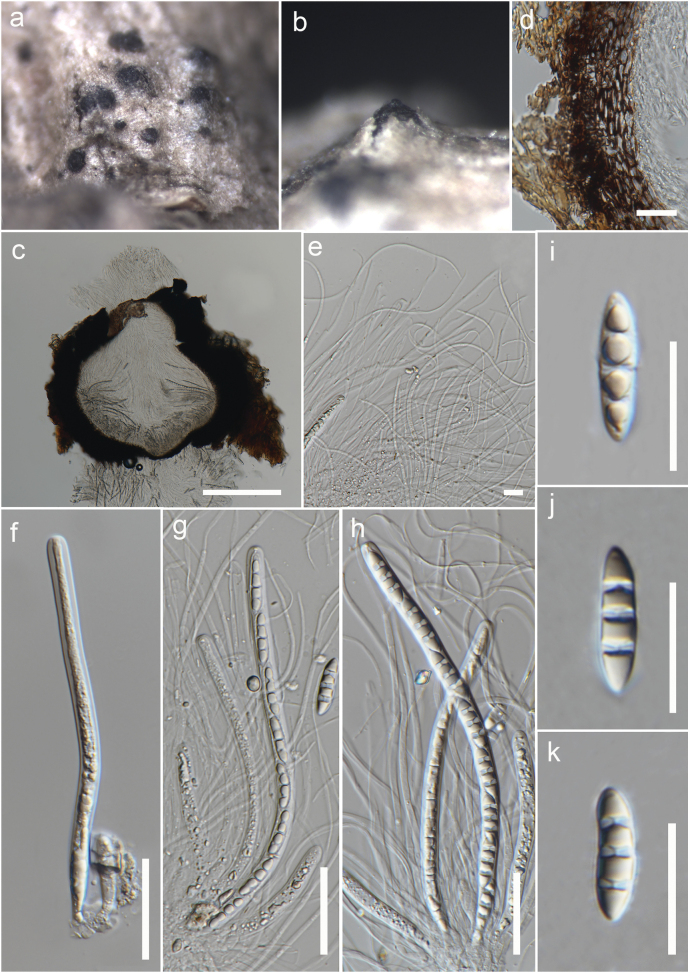
*Melomastiaoleae* (MFLU 23-0144) **a** ascomata on the stem of *Durantaerecta***b** an ascoma erumpent through the host tissue **c** vertical section of an ascoma **d** peridium **e** pseudoparaphyses **f–h** asci **i–k** ascospores. Scale bars: 200 µm (**c**); 20 µm (**d, i–k**); 5 µm (**e**); 30 µm (**f–h**).

##### Culture characteristics.

Colonies on PDA reaching 20 mm diam. in 3 weeks at 25 °C. Culture from above circular, regular, entire margin, dense, white; reverse pale brown.

##### Material examined.

Thailand • Chiang Rai, Mae Fah Luang University, Mueang, Tha Sut; on dead stems and twigs of *Durantaerecta*, 30 Nov 2021, V. Thiyagraja DB 184, Herbarium material MFLU 23-0144, living culture MFLUCC 23-0086.

##### Notes.

The isolate MFLUCC 23-0086 in the present study is basal to all the strains of *M.oleae* (Fig. 3). There was 0.2% (2/986 bp) nucleotide difference in SSU, while 0.9% (8/872 bp) in LSU and 0.1% (1/894 bp) in *tef1-α* between strain MFLUCC 23-0086 and the type strain (CGMCC3.20619) of *M.oleae*. Isolate MFLUCC 23-0086 is morphologically similar to *M.oleae* in terms of ascomata which appear as cone-shaped on the host surface, filiform and unbranched pseudoparaphyses, cylindrical, pedicellate asci and uniseriate, fusiform, 3-septate ascospores. However, the asci and ascospore sizes of the type of *M.oleae* are larger than our collection. Furthermore, the peridium of strain MFLUCC 23-0086 is observed as comprising thick-walled, brown cells of *textura angularis* to *textura epidermoidea* in the outer region and compressed, hyaline cells in the innermost region (Fig. 7). The type of *M.oleae* is reported to have a peridium with an outer thick, carbonaceous layer and the inner one made up of 5–6 layers of hyaline cells of *textura angularis* to *textura prismatica* (Li et al. 2022). Despite these morphological differences, MFLUCC 23-0086 is recognized as *M.oleae* as there is insufficient genetic variation to distinguish it as a different species (Maharachchikumbura et al. 2021; Pem et al. 2021). Furthermore, the morphological differences may be due to different hosts and environmental conditions. Therefore, *M.oleae* is herein introduced as a new record from *Durantaerecta* in Thailand.

## ﻿Discussion

In the present study, the familial description of the sexual morph of taxa in Pleomonodictydaceae is emended to include *Robiniigena* gen. nov. and *Inflatispora*, as both share several common morphological characteristics. *Robiniigena* also shares similar features with taxa belonging to the sister clade Morosphaeriaceae (Hongsanan et al. 2020). *Robiniigena* has cellular pseudoparaphyses, similar to *Aquihelicascus* and *Neohelicascus* taxa (Dong et al. 2020) and short-pedicellate asci resembling those of *Aquilomyces* and *Clypeoloculus* (Tanaka et al. 2015). *Robiniigena* also has hyaline ascospores with a gelatinous sheath similar to species of *Aquilomyces*, *Clypeoloculus*, and *Morosphaeria* (Suetrong et al. 2009; Tanaka et al. 2015; Devadatha et al. 2018). *Robiniigena* differs from Morosphaeriaceae species in that its ascomata are not covered with brown hyphae, as in *Aquilomyces* and *Clypeoloculus* (Tanaka et al. 2015). It has short-pedicellate asci while taxa of *Aquihelicascus* comprise asci with long pedicels (Dong et al. 2020). Ascospores are hyaline in *Robiniigena* while those of several *Helicascus* and *Neohelicascus* taxa are brown (Preedanon et al. 2017; Zeng et al. 2018; Dong et al. 2020). The introduction of *Robiniigena* and the inclusion of the *incertae sedis Inflatispora* in Pleomonodictydaceae indicates that the family is under constant taxonomic review. Further taxon sampling will undoubtedly give a better insight into the family as well as its relationship with other families in Massarineae.

*Robiniigenahyalinospora* was isolated from dead branches of *Robiniapseudoacacia* in Italy. Similarly, other fungi have been reported from the same host in Italy. *Cladosporiumnigrellum* and *Camarosporidiellamirabellensis*, for instance, were isolated from dead or decorticated branches of *R.pseudoacacia* (Bensch et al. 2012; Wanasinghe et al. 2017). *Dothidotthiarobiniae* was also retrieved from the same host as a saprobe, but from the Russian Federation (Senwanna et al. 2019). *Camarosporidiellaelongata* (syn. *Cucurbitariaelongata*) and *Massariaanomia* (syn. *Aglaosporaprofusa*) were pathogenic on *R.pseudoacacia* in Greece (Michalopoulos-Skarmoutsos and Skarmoutsos 1999). Other fungi from the class Sordariomycetes have also been recorded from this host. For instance, *Colletotrichumnymphaeae* was identified as a pathogen of *R.pseudoacacia* in Japan (Yamagishi et al. 2016), while *Diaportheoncostoma* was isolated from dead twigs of *R.pseudoacacia* (even though the species was also reported as a weak parasite) in Bulgaria (Stoykov 2012). These findings indicate that *Robiniapseudoacacia* hosts a diverse range of fungi with different lifestyles, across a large geographical area.

In this study, we have also discovered and reported the previously unknown sexual morph of *Ampelomycesquisqualis* (Wijayawardene et al. 2021). *Ampelomyces* comprises 16 species (Species Fungorum 2024), but sequence data are available for only *Ampelomycesquisqualis*. The inclusion of the genus in Phaeosphaeriaceae has been accepted by Phookamsak et al. (2014) based on the type species *A.quisqualis* (CBS 129.79). However, the latter strain is not the type or a verified strain of *A.quisqualis* (de Gruyter et al. 2009; Phookamsak et al. 2014; Vu et al. 2019; Hongsanan et al. 2020). Herein, we have included several representative strains of *A.quisqualis* (from NCBI) in our phylogeny, and they all cluster in a clade, but with three subclades (Fig. 2). Our collection MFLU 23-0142 grouped with *A.quisqualis* strains (AMP, Chillan, BRIP 72107, and CBS 133.32) in subclade A. A recent study by Huth et al. (2021) compared the draft genome assemblies for *A.quisqualis* strains (BRIP 72107 and HMLAC 05119; NCBI names) and revealed that the two strains are not conspecific. In the present study, strain HMLAC 05119 clustered with other *A.quisqualis* strains in subclade B, while *A.quisqualis* BRIP 72107 is nested in subclade A (Fig. 2). The presence of the subclades potentially indicates that all the strains may not actually be *A.quisqualis*; they probably belong to several different lineages in *Ampelomyces*. Several previous studies have also made such observations (Lee et al. 2007; Park et al. 2010; Angeli et al. 2011; Liyanage et al. 2018). Therefore, it is vital that *A.quisqualis* is recollected and the type sequenced to resolve these ambiguities. Until this taxonomic uncertainty is resolved, strain MFLU 23-0142, collected in the present study, is referred to *A.quisqualis*, as the sexual morph. In another scenario, *Ampelomyces* has been reported to cluster with *Neosetophoma* taxa in other phylogenetic studies (Phookamsak et al. 2014; Tibpromma et al. 2017; Hyde et al. 2018; Hongsanan et al. 2020). Until *Ampelomyces* is typified, its generic status, as well as its phylogenetic placement (including that of ‘*A.quisqualis*’ strains), remain uncertain.

*Melomastiamaolanensis* was originally described as a saprobe on dead branches of an undetermined host from Guizhou, China (Zhang et al. 2017), while *M.oleae* was collected from dead branches of *Oleaeuropaea* at the foot of a mountain or mountainside in Sichuan, China (Li et al. 2022). As mentioned above, the strains collected in this study were similarly obtained from terrestrial habitats, although from different geographical locations and hosts. Some *Melomastia* taxa, such as *M.aquatica*, *M.neothailandica*, and *M.thailandica* have been collected from aquatic habitats as saprobes (Hyde 1992; Hyde et al. 2016; Dayarathne et al. 2020). *Melomastiaseptemseptata*, a recently introduced novel taxon based on morphological support, and characterized by dark green ascomata and multi-septate ascospores, has been found to occur on living tree bark (corticolous) in the terrestrial environment (Naziazeno and Aptroot 2023). *Melomastiadistoseptata* has been reported from both terrestrial and freshwater habitats (Hongsanan et al. 2020; Boonmee et al. 2021). It is valuable to document new records of existing species, either from new geographical locations, habitats, hosts, or with different lifestyles; this enables a better understanding of the diversity and ecology of the fungal species, host jumping, and adaptations of fungi to various environmental conditions (Hyde et al. 2020; Chethana et al. 2021).

The phylogeny of the genera *Dyfrolomyces* and *Melomastia* has been confusing and not truly resolved. *Dyfrolomyces* was introduced by Pang et al. (2013) as part of a study on marine *Saccardoella* species, a genus variously classified in Clypeosphaeriaceae, Xylariales (Barr 1989), Pleurotremataceae, Xylariales (Barr 1994), and ‘Unitunicate Ascomycota genera *incertae sedis* (Jones et al. 2009). Suetrong et al. (2009) earlier established that *Saccardoellarhizophorae* did not show any affinities to members of the Sordariomycetes (Maharachchikumbura et al. 2015), but grouped within the Dothideomycetes, although it did not cluster with any known families or orders in the class. Pang et al. (2013) confirmed the monophyly of *S.rhizophorae* and the new marine fungus *S.tiomanensis*, in a sister clade to Acrospermaceae (Dothideomycetes) and separately from *S.montellica*, the type species of the genus referred to Sordariomycetes at that time. Pang et al. (2013), therefore, introduced a new genus *Dyfrolomyces* to accommodate *S.rhizophorae*, *S.tiomanensis* as well as *S.mangrovei* and *S.marinospora*, and also established the family Dyfrolomycetaceae in the order Dyfrolomycetales (Hyde et al. 2013). Later, Maharachchikumbura et al. (2016) synonymized Dyfrolomycetaceae under Pleurotremataceae. Norphanphoun et al. (2017) noted that *Melomastiaitalica* and *Dyfrolomycesmaolanensis* formed a sister clade to five *Dyfrolomyces* species, and referred *D.maolanensis* to *Melomastia*. They concluded that *Dyfrolomyces*, *Pleurotrema*, and *Melomastia* belonged in the Pleurotremataceae, and shared many features in common, but considered *Melomastia* and *Dyfrolomyces* “as distinct genera”. It is important to record that there were no sequences of *Melomastia* available when Pang et al. (2013) introduced the genus *Dyfrolomyces*.

In the study by Li et al. (2022), *Dyfrolomyces* and *Melomastia* species clustered together, which led to *Dyfrolomyces* and its taxa being synonymized under *Melomastia*. In the current phylogenetic study, *Melomastia* species form three clades (A, B, and C; Fig. 3). *Melomastiatiomanensis* (type species of *Dyfrolomyces*) and *M.chromolaenae* form a basal clade (clade C) with 100% ML BS, 1.00 BYPP support. Although they share common features with several *Melomastia* species, especially in their short-pedicellate, apically rounded asci, and multi-septate ascospores, they mainly differ from the other *Melomastia* taxa in their distinct spindle-shaped ascospores with tapering narrow ends (Pang et al. 2013; Mapook et al. 2020). The status of *Dyfrolomyces* is therefore justified for *M.tiomanensis* and *M.chromolaenae* based on morphological and phylogenetic evidence, as is equally reported by Kularathnage et al. (2023).

Meanwhile, the remaining *Melomastia* taxa (with molecular data available) form two clades (A and B; Fig. 3). Morphological delineation between the two clades is challenging since the taxa have overlapping features. The type species, *M.mastoidea* is characterized by 2-septate, ovoid ascospores with rounded ends, which are surrounded by a mucilaginous sheath, but it lacks sequence data. Kularathnage et al. (2023) have classified clade B as *Melomastiasensu stricto* since the taxa in that clade share most of the ascospore features with *M.mastoidea*. Clade A, which has been termed as *Melomastiasensu lato*, contains taxa whose ascospore morphology is less similar to that of *M.mastoidea*. However, this classification may not be totally accurate since *M.fusispora* in clade A also has a gelatinous sheath (Li et al. 2022). Taxa such as *M.septata*, *M.maolanensis*, and *M.sichuanensis*, which are accommodated in clade B, have 3-septate ascospores, similar to *M.oleae*, *M.distoseptata*, *M.fusispora* and *M.winteri* in clade A (Zhang et al. 2017; Hongsanan et al. 2020; Li et al. 2022; Hyde et al. 2023). Furthermore, the shape of the ascospores in both clades A and B are cylindrical, ellipsoidal to fusoid or fusiform, with rounded to acute ends. Clades A and B can indeed be indicative of two or more different genera. Moreover, *M.phetchaburiensis* and *M.sinensis*, which have been treated as uncertain species in *Melomastia* by Dong et al. (2023), may represent different genera. However, the definite classification of these taxa will be possible upon the availability of molecular data for the type species, *M.mastoidea*. Also, it could be that other characters are still underplayed. For instance, none of the taxa in either clade A or B have their asexual morphs reported so far. Therefore, with the collection of more taxa in both clades, a better resolution of their position in Pleurotremataceae will be possible. Moreover, employing more genetic markers in the phylogenetic analysis of Pleurotremataceae may provide a better and more accurate picture of the taxa in the family.

The phylogeny of *Melomastia* and *Dyfrolomyces* has long been challenging in that no sequences of *Melomastia* species were available when Pang et al. (2013) introduced *Dyfrolomyces*. Subsequent phylogenetic studies have failed to undertake an analysis of pairwise distances between the two genera or of the family Pleurotremataceae. Likewise, base pair differences between *Melomastia* species might have clarified their taxonomy if the guidelines of Jeewon and Hyde (2016) and Maharachchikumbura et al. (2021) had been followed, especially when the *Dyfrolomyces* species were synonymized by Li et al. (2022).

It is also to be noted that the phylogram in Fig. 1 involves the taxa *Pleomonodictyscapensis* and *Pl.descalsii* which are clustered together but with no robust phylogenetic segregation. *Pleomonodictysdescalsii* was introduced principally based on its smaller conidial size as compared to that of *Pl.capensis* (Hernández-Restrepo et al. 2017). However, Bao et al. (2021), while introducing a novel collection for *Pl.capensis*, noticed that the conidial size of their new collection closely matched that of *Pl.descalsii*, and there was also no remarkable difference vis-à-vis the other asexual features. This indicates that *Pl.capensis* could be conspecific with *Pl.descalsii* and this is equally being shown in the present phylogeny (Fig. 1). Additional morpho-phylogenetic studies are necessary to solve this taxonomic uncertainty.

## Supplementary Material

XML Treatment for Pleomonodictydaceae

XML Treatment for Inflatispora

XML Treatment for Robiniigena

XML Treatment for Robiniigena hyalinospora

XML Treatment for Ampelomyces quisqualis

XML Treatment for Melomastia maolanensis

XML Treatment for Melomastia oleae

## ﻿Additional information

### Author ORCIDs

Digvijayini Bundhun https://orcid.org/0000-0002-0790-215X

E. B. Gareth Jones https://orcid.org/0000-0002-7286-5471

Ruvishika S. Jayawardena https://orcid.org/0000-0001-7702-4885

Erio Camporesi https://orcid.org/0000-0002-2801-9462

Dhanushka N. Wanasinghe https://orcid.org/0000-0003-1759-3933

Indunil C. Senanayake https://orcid.org/0000-0002-7165-2394

Vinodhini Thiyagaraja https://orcid.org/0000-0002-8091-4579

Kevin D. Hyde https://orcid.org/0000-0002-2191-0762

### Data availability

All of the data that support the findings of this study are available in the main text.
